# Chitosan-Based Coatings for Orthodontic Appliances: Antimicrobial Properties, Potential Ion-Release Mitigation, and Clinical-Translation Perspectives

**DOI:** 10.3390/jfb17080371

**Published:** 2026-08-01

**Authors:** Marcin Mikulewicz

**Affiliations:** Department of Dentofacial Orthopaedics and Orthodontics, Division of Facial Abnormalities, Medical University of Wrocław, Krakowska 26, 50-425 Wrocław, Poland; marcin.mikulewicz@umw.edu.pl

**Keywords:** chitosan, orthodontic appliances, antibacterial coating, nickel release, corrosion resistance, biodegradability, biocompatibility, clinical translation

## Abstract

Fixed orthodontic appliances promote biofilm-mediated enamel demineralization and release metallic ions, motivating surface strategies that are intrinsic to the device rather than dependent on patient compliance. Chitosan, a biodegradable polycationic biopolymer, has been proposed as a multifunctional coating. This narrative review critically appraises the evidence for chitosan-based coatings on orthodontic appliances across three pillars—antimicrobial performance, ion-release/corrosion mitigation, and clinical translation—with explicit calibration of evidentiary strength. The evidence base is heterogeneous and of markedly uneven quality across the three pillars, and the conclusions below are weighted accordingly. Consistent with its narrative design, the literature was surveyed for critical synthesis rather than exhaustively, without formal eligibility screening, risk-of-bias appraisal, or quantitative pooling. Antimicrobial efficacy is the best-supported pillar, consistent in vitro and now extended by two short in vivo randomized trials, although all endpoints are microbiological surrogates rather than white spot lesion outcomes. Ion-release mitigation remains a plausible but insufficiently demonstrated effect: it is supported only indirectly, through electrochemical corrosion proxies on predominantly implant substrates, and no study quantifies ion-release reduction from a coated appliance. Coating durability under combined enzymatic and mechanical load—and its effect on friction—remains largely uncharacterized. Chitosan coatings are promising but translationally immature; durability, not antimicrobial potency, is the rate-limiting barrier. Defined clinical-endpoint and appliance-level ion-release studies are required.

## 1. Introduction

Fixed orthodontic appliances impose a sustained ecological burden on the oral cavity. Brackets, bands, and archwires create stagnation zones that resist mechanical cleansing, shift the supragingival biofilm toward acidogenic and aciduric species, and depress plaque pH at the enamel interface. The clinical consequence is well quantified: a recent systematic review and meta-analysis reported a pooled white spot lesion (WSL) prevalence of approximately 55% and an incidence of approximately 34% among orthodontic patients, with conventional fixed appliances conferring roughly 4.7-fold higher odds of WSL than other appliance types and seven-fold higher odds than no treatment [1]. Demineralization can begin within weeks of bonding, with lesion development influenced by adhesive selection and plaque accumulation at bracket margins [2]. Because WSLs are frequently irreversible at the esthetic level, a corrective intervention can acquire an iatrogenic dimension.

A second, mechanistically distinct liability arises from the appliance materials themselves. Stainless steel and nickel–titanium (NiTi) components undergo electrochemical corrosion in the fluctuating-pH, chloride-rich, enzymatically active oral environment, releasing nickel, chromium, and other ions. Quantitative inductively coupled plasma spectrometry confirms measurable intraoral ion release across appliance alloys [3], and combined in vitro–in vivo modeling has been used to estimate the delivered dose over a treatment course [4]. Fluoride-containing agents—paradoxically, the same agents deployed against demineralization—can markedly accelerate nickel and titanium release from NiTi [5]. Although the released quantities usually remain below acute-toxicity thresholds, nickel is a prevalent contact allergen, and longitudinal data show that a non-trivial fraction of patients exhibit or develop nickel sensitization during treatment, without a simple dose–response relationship to salivary ion load [6]. Attenuating both bacterial colonization and ionic egress at the appliance surface is therefore a coherent, dual-target design objective.

Conventional preventive adjuncts—fluoride varnishes and rinses, chlorhexidine, and dietary counseling—are compliance-dependent and transient, and they do not address ion release at all. This motivates functionalizing the appliance surface itself, so that antimicrobial and barrier performance become intrinsic to the device rather than contingent on patient behavior. Among candidate coating chemistries, chitosan—the partially deacetylated derivative of chitin—is distinctive in addressing more than one objective simultaneously. Its protonated amino groups confer a polycationic character that perturbs negatively charged microbial envelopes, producing intrinsic, leach-independent antibacterial activity whose magnitude scales with the degree of deacetylation, molecular weight, and local pH [7,8,9]. The same macromolecule is film-forming, biocompatible, inexpensively recoverable from marine waste streams, and enzymatically biodegradable [10,11]. That final property is double-edged: biodegradability underwrites biocompatibility yet simultaneously threatens the durability of a coating expected to survive 18–24 months of intraoral loading—a tension examined explicitly in Section 5.

Conceptually, a chitosan coating could serve the fixed appliance in three registers: as an antimicrobial layer limiting biofilm-mediated demineralization; as a physical and electrochemical barrier attenuating metal-ion release; and as a biocompatible, potentially bioactive interface amenable to compositing with therapeutic agents. These registers are unevenly supported by evidence, and the present review is deliberately organized to reflect that asymmetry rather than to obscure it.

Existing reviews approach adjacent territory without occupying this niche. Ahuja et al. meta-analyzed nanoparticle antibacterial coatings on orthodontic materials but treated chitosan as one agent among several and addressed only the antimicrobial endpoint [12]; Wang et al. surveyed antibacterial coatings for appliances at the mechanistic level without integrating corrosion or translation [13]; Li et al. reviewed surface modification of biomedical NiTi—including corrosion and ion release—but did so neither chitosan-specifically nor within the orthodontic appliance context [14]; and He et al. surveyed nanotechnology across orthodontics broadly [15]. No current synthesis is at once chitosan-specific and appliance-specific while integrating the antimicrobial, ion-release/corrosion, and clinical-translation dimensions within a single, critically appraised framework.

Accordingly, this narrative review synthesizes the evidence for chitosan-based coatings on orthodontic appliances across three pillars—antimicrobial properties, ion-release/corrosion mitigation, and clinical translation—while maintaining explicit calibration of evidentiary strength. We treat the antimicrobial evidence as the empirical core; the ion-release-mitigation claim as a mechanistically plausible inference drawn from corrosion-proxy and implant-derived data rather than a demonstrated property of coated appliances; and clinical translation as a gap-and-roadmap analysis. The durability–biodegradability tension and coating–friction interactions, routinely omitted from more optimistic surveys, are treated as central rather than peripheral.

### Search Strategy

This is a narrative review, not a systematic review, with the stated search intended more for retrieval and critical synthesis than for exhaustive collection. The distinction is material to interpretation: the synthesis that follows is selective and interpretive by design, and none of its conclusions should be read as carrying systematic-review-level certainty. We searched the PubMed/MEDLINE, Scopus, and Web of Science databases from inception to July 2026 for combinations of chitosan, coating, orthodontic, bracket, archwire, aligner, antibacterial/antimicrobial, corrosion, nickel/ion release, and biocompatibility keywords. Primary studies (in vitro, in vivo) and reviews regarding the use of chitosan or chitosan-composite coatings on orthodontic appliances or other comparable biomaterials made of metal were included. Studies were retained when they reported primary data or structured synthesis on chitosan or chitosan-composite coatings applied to orthodontic appliances or to comparable metallic biomaterials (implant alloys, NiTi, stainless steel) and were set aside when chitosan was used in a non-coating context (for example bulk hydrogels or drug-delivery particles unrelated to surface modification) or when the substrate had no orthodontic or metallic-biomaterial analog. To reduce the risk that relevant appliance-level work was overlooked, the reference lists of the retrieved reviews and primary studies were additionally hand-searched (backward citation tracking), and the specific observation that no ICP-OES/ICP-MS ion-release measurement exists for a chitosan-coated orthodontic appliance was re-checked with targeted database queries before being reported as ‘not identified’. Sources were selected based on the authors’ judgment of relevance and methodological salience; no eligibility criteria, screening algorithm, risk-of-bias tool, or quantitative data synthesis was utilized, and this review was not registered. The cited sources were primarily published within the past five years; other sources were cited when they established a foundational concept, mechanism, or method. The article is structured and reported according to the SANRA (scale for the assessment of narrative review articles) guidelines. The claims that a particular study has not been conducted, such as the lack of an ICP-OES/ICP-MS ion-release study at the level of the orthodontic appliance using a chitosan-coated wire, are made in light of the stated but nonspecific search and should be considered ‘not identified’ rather than as evidence that it does not exist.

## 2. Chitosan: Structure–Property Basis for Surface Coatings

Chitosan is not a single compound but a family of linear polysaccharides—copolymers of N-acetyl-D-glucosamine and D-glucosamine—produced by partial deacetylation of chitin, the structural polysaccharide of crustacean and fungal biomass [10]. Two compositional variables govern nearly all functional behavior relevant to a coating: the degree of deacetylation (DD), which sets the surface density of primary amino groups, and the molecular weight (MW), which sets chain length, solution viscosity, and film cohesion. Because both are tunable during extraction and processing, chitosan is more accurately treated as a formulation space than as a fixed material—a distinction with direct consequences for coating reproducibility and for the comparability of studies that report neither parameter [10].

The antimicrobial function originates in protonation of the glucosamine amino groups below their apparent pKa (~6.3–6.5). The resulting polycation adsorbs electrostatically to anionic constituents of the bacterial envelope—teichoic acids in Gram-positive organisms and lipopolysaccharides and phospholipids in Gram-negative organisms—perturbing membrane integrity, raising permeability, and provoking leakage of intracellular contents [7,8]. Secondary mechanisms include chelation of trace metals and essential nutrients and, for low-MW fractions, penetration and binding to intracellular targets. Two structure–property regularities follow and are experimentally supported: antibacterial potency rises with DD as cationic charge density increases [8,9]; and the dominant mode shifts with MW, with high-MW chains forming a surface-enveloping, agglutinating layer while low-MW chains act more intracellularly [7]. Critically, activity is strongly pH-dependent and attenuates near neutrality. Resting salivary pH sits close to chitosan’s pKa, so a coating’s polycationic activity is only partially expressed at rest and is up-regulated precisely during the acidogenic challenges that drive demineralization—a mechanistically favorable, if incompletely characterized, coupling.

Translating this chemistry onto an appliance requires a deposition route that yields an adherent, uniform film on a passivated metallic substrate. Reported approaches span solvent casting and dip-coating—used, for example, to deposit non-crosslinked chitosan directly onto brackets [16]—through electrophoretic deposition (EPD), which exploits the polycation’s charge to co-deposit chitosan with functional particles onto conductive substrates such as orthodontic bands [17], to electrodeposition of chitosan–nanoparticle composite films on titanium [18] and stainless steel [19]. EPD suits appliance geometries because the film thickness and composition are controllable through applied potential and suspension chemistry and because ceramic or metallic nanofillers can be entrained in a single step [19,20]. The recurring engineering obstacle is adhesion: chitosan bonds poorly to inert passivated alloys, so coating adhesion and mechanical integrity are themselves active experimental variables rather than solved problems [20]. Surface pretreatment or covalent crosslinking is typically required, and crosslinking is a genuine trade-off rather than a free improvement—it raises hydrolytic and mechanical stability at the cost of consuming the amino groups responsible for antibacterial activity and, with some agents, of introducing cytotoxicity.

A second design axis is compositing. Chitosan functions readily as a polymeric matrix that disperses and moderates the release of antibacterial or remineralizing agents—silver, zinc oxide, copper, bioactive glass, hydroxyapatite, and carbon-based nanomaterials—so that its intrinsic polycationic activity is augmented by a second, filler-derived mechanism [19,20,21]. This matrix role underlies most of the appliance-directed evidence examined in Section 3 and Section 4 and it complicates causal attribution: when a chitosan–nanoparticle composite performs well, the polymer’s own contribution is routinely entangled with the filler’s, and few studies include the uncoated-chitosan control needed to separate them.

The property that most distinguishes chitosan from inert coatings is its biodegradability. Chitosan is hydrolyzed in vivo by lysozyme and other glycosidases at a rate that rises as DD falls, yielding non-toxic oligosaccharides and underpinning a generally favorable biocompatibility profile [10,22]. For a resorbable scaffold this is an asset; for a barrier coating expected to persist across an 18–24-month course under combined mechanical and enzymatic load, it is a liability that the optimistic literature seldom quantifies. Section 5 returns to this durability–biodegradability tension as the defining unresolved problem of the field.

## 3. Antimicrobial Performance on Orthodontic Appliances

Antimicrobial activity is the pillar on which the entire chitosan-coating rationale rests and the only one currently supported by in vivo human data. The mechanistic basis established in Section 2—leach-independent, contact-mediated membrane disruption by a surface-bound polycation—predicts that a chitosan layer should suppress colonization at the appliance surface without depending on a diffusible agent that is consumed or washed out. The available evidence, spanning brackets, molar bands, archwires, and 3D-printed aligners, is broadly consistent with that prediction in vitro, and two recent randomized trials extend it, cautiously, to the mouth. The evidence must nonetheless be read against a hard distinction: every study to date measures a microbiological surrogate—colony-forming units (CFUs) or biofilm mass on the appliance—rather than the clinical endpoint that motivates the field, namely white spot lesion (WSL) incidence [1]. Reduced surface CFU is a necessary but not sufficient condition for caries prevention; the causal step from the former to the latter remains assumed, not demonstrated.

At the highest available evidence tier, two in vivo randomized controlled trials (RCTs) exist. Patel et al. coated 0.016 in NiTi archwires with chitosan nanoparticles by ionic gelation and compared them with uncoated controls in a 30-patient split-mouth design (60 wires, allocation-concealed), retrieving the wires after six weeks and quantifying adherent bacteria [23]. Coated wires carried markedly lower burdens of both *Streptococcus mutans* (0.12 ± 0.38 vs. 1.82 ± 0.69 × 10^6^ CFU/mL) and *Lactobacillus acidophilus* (0.036 ± 0.06 vs. 1.96 ± 0.65 × 10^6^ CFU/mL; both *p* < 0.001)—reductions of approximately 93% and 98%, respectively (calculated from the reported CFU means [23]). Alhuwaizi and Saloom incorporated 3% chitosan nanoparticles into 3D-printed clear aligners and assessed antibiofilm activity against *S. mutans* after one and two weeks of intraoral wear (*N* = 32 total) [24]. After two weeks, the chitosan group showed significantly lower plaque and bleeding indices (plaque index 4.61 ± 1.22 vs. 16.16 ± 5.26; bleeding on probing 3.83 ± 1.03 vs. 5.35 ± 3.03) and reduced CFUs (effect size > 0.9), with the important nuance that efficacy was weaker against a clinical isolate than against the reference ATCC strain (week-2 effect size 0.621 vs. 0.953)—a reminder that laboratory reference organisms may overstate performance against native oral flora. Both trials are genuine advances, but both are short (six and two weeks against a treatment course of 18–24 months) both rely on surrogate endpoints, and neither addresses whether the coating survives function.

Below the clinical tier, the in vitro and animal evidence is more abundant and, for present purposes, more mechanistically informative. The cleanest demonstration that chitosan itself—not a co-deposited filler—drives the effect comes from Wang et al., who coated brackets with non-crosslinked chitosan by immersion and freeze-drying and showed dose-dependent inhibition of *S. mutans* biofilm formation rising from 48.5% to 99.6% across coating concentrations of 1.0–10 mg/mL, with free chitosan additionally eradicating mature biofilm (≈99.8%) [16]. Most other appliance studies test composites, in which chitosan serves as a polymeric matrix for a second antibacterial agent. Fatima et al. deposited a chitosan–zinc oxide nanocomposite on molar bands by electrophoretic deposition and reported significant activity against *S. mutans* that peaked with zinc release on day six—dual evidence that also bears on Section 4, since the same coating shifted corrosion potential toward nobler values and lowered corrosion current density [17]. Liu et al. coated brackets with carboxymethyl-chitosan/xylitol carbon dots (adhered via polydopamine), achieving an ≈80% antibacterial rate against *S. mutans* together with enamel remineralization and acceptable L929 cytocompatibility, corroborated in a murine infection model [25]. Tawakal et al. compared silver and silver–chitosan nanoparticle coatings on ceramic and metallic brackets, confirming strong anti-*S. mutans* activity but also flagging coating-dependent increases in static friction and surface roughness on metallic substrates [26]—a functional trade-off developed in Section 5.

Placed against non-chitosan coatings, chitosan’s comparative advantage is qualitative rather than one of raw potency. Silver coatings on fixed appliances produce consistent, well-cataloged reductions in bacterial adhesion and biofilm across brackets, archwires, and microimplants [27], and silver-based nanocomposite layers have been radio-frequency sputtered onto NiTi archwires to yield smooth, antibacterial surfaces [28]; ceramic coatings such as zirconium oxide applied to stainless steel and NiTi wires show comparable antibacterial activity in vitro [29]. The systematic review and meta-analysis by Ahuja et al. found significant antibacterial gains across this whole class of agents, chitosan among them, but with substantial between-study heterogeneity and no consensus protocol [12]. Biofilm formation is, moreover, sensitive to the archwire’s surface and coating chemistry itself [30], underscoring that coating composition modulates not only antibacterial action but baseline microbial adhesion. Chitosan’s distinguishing properties are its intrinsic, non-leaching mechanism, its biodegradability-linked biocompatibility, and its dual role as an active agent and a carrier matrix. A further, clinically salient distinction is stimulus-responsiveness. Silver and other leaching agents release their active species in a broadly pH-independent, monotonically depleting manner, whereas chitosan’s contact killing derives from amino-group protonation and is therefore up-regulated at the low pH of an acidogenic biofilm, concentrating antibacterial action precisely when and where demineralization risk is greatest. This pH-gated, ‘on-demand’ behavior is a potential advantage over pH-independent coatings, although it has not yet been deliberately exploited or quantified in appliance studies. That last property is also the principal confounder in the appliance literature: because most appliance studies evaluate chitosan–nanoparticle composites without a chitosan-only comparator arm, the polymer’s independent antibacterial contribution cannot be isolated from the filler’s; factorial designs that separate the two remain rare, with Wang et al. [16] a notable exception.

Three methodological limitations bound the strength of this pillar. First, endpoint surrogacy: no study measures WSL or caries incidence, , so efficacy against the outcome clinicians care about is inferred rather than demonstrated.. Second, heterogeneity and under-reporting of the determinants Section 2 identified as decisive—degree of deacetylation, molecular weight, coating thickness, and crosslinking—which precludes meaningful cross-study synthesis and is the explicit reason the one available meta-analysis declined to pool a chitosan-specific estimate [12]. Third, model artificiality: static in vitro assays omit salivary flow, pellicle formation, dietary pH cycling, and the mechanical abrasion of ligation and sliding, all of which plausibly degrade real-world performance and none of which the short in vivo trials ran long enough to capture. Publication bias toward positive results compounds all three.

The defensible conclusion is narrow but real: chitosan and chitosan-composite coatings reliably reduce bacterial colonization of orthodontic appliances in vitro, and two short RCTs now show that a chitosan coating can lower surface bacterial load and gingival indices in vivo. What no study yet establishes is that this surrogate benefit translates into fewer WSLs, or that the coating retains activity across a full treatment course under mechanical load—questions that Section 5 and Section 6 argue are the field’s true rate-limiting steps (Table 1).

## 4. Ion-Release/Corrosion Mitigation and Biocompatibility

This is the pillar where the gap between what the title asserts and what the literature demonstrates is widest, and the section is written to make that gap explicit rather than to paper over it. The claim of ion-release mitigation implies a causal chain with four links: a chitosan coating (i) forms a barrier that (ii) improves corrosion resistance, which (iii) reduces metal-ion egress, which (iv) lowers the biological risk to the patient. Link (ii) has direct experimental support; link (iii) is inferred from electrochemical proxies rather than measured on orthodontic appliances; and link (iv) is complicated by evidence that sensitization is not a simple function of ionic dose. Distinguishing these links is the analytical work of this section.

The problem the coating would address is real and quantified. Uncoated appliance alloys corrode in artificial saliva and release nickel and chromium, with NiTi and Cr–Co wires releasing significantly more than stainless steel under identical conditions [3]; comparable ICP-based release has been documented for stainless steel pediatric appliances [31]. In vivo, release is measurable in saliva, serum, and hair, and combined in vitro–in vivo modeling has been used to estimate the delivered dose over a treatment course, with transfer coefficients quantifying the fraction of released ions reaching biological compartments [4]. The oral environment aggravates the baseline: acidulated-fluoride agents can raise nickel and titanium release from NiTi by orders of magnitude and degrade the alloy’s superelasticity through martensitic transformation [5]. The biological endpoint that justifies concern, however, resists a clean dose–response account—longitudinal data show nickel sensitization in roughly one-third of orthodontic patients without a simple relationship to salivary ion load [6]. This matters for the review’s logic: even a coating that demonstrably reduced ion release would not straightforwardly guarantee reduced sensitization, so link (iv) cannot be assumed from link (iii).

Direct evidence that a chitosan coating mitigates corrosion on an orthodontic appliance reduces to a single dataset. Fatima et al.’s chitosan–ZnO nanocomposite on molar bands shifted the corrosion potential in the nobler direction (−0.6966 → −0.5949 V) and lowered corrosion current density (7.81 → 4.04 µA/cm^2^), evidencing improved corrosion resistance on an actual appliance [17]. The only other appliance-level corrosion evidence uses a non-chitosan coating: a polydopamine–graphene-oxide layer on NiTi archwires that prolonged the corrosive-medium diffusion path and reduced nickel dissolution while remaining cytocompatible [32]. Both are electrochemical or dissolution proxies; neither is an inductively coupled plasma quantification of ionic release from a chitosan-coated wire under oral-simulating conditions.

The remainder of the supporting evidence is drawn from implant and tissue-engineering contexts and is flagged as such throughout. Chitosan-composite coatings improve the corrosion behavior of NiTi and related alloys across several such studies: a chitosan/Cu-nanoparticle multilayer on NiTi enhanced corrosion resistance (reduced corrosion current density), was non-cytotoxic to MG-63 osteoblast-like cells, and released copper linearly over seven days [33]; a chitosan-containing composite on additively manufactured (LPBF) NiTi likewise reduced corrosion current density while maintaining >100% relative cell viability and >70% antibacterial efficacy [34]; an electrodeposited chitosan–gold-nanoparticle film on titanium reported ~98% corrosion inhibition efficiency in Hanks’ solution [18]; and chitosan-composite coatings on 316L stainless steel and titanium, deposited by electrophoretic deposition, combined corrosion resistance with cytocompatibility meeting ISO 10993-5 [19,20]. Systematic and narrative syntheses of chitosan-modified titanium implants [21] and of NiTi surface modification broadly [14] reach the same qualitative conclusion. The consistent finding across this body of work is that chitosan-based composite layers act as corrosion-attenuating barriers on metallic biomaterials—but every one of these substrates is an implant, none is an orthodontic appliance under oral loading, and corrosion current density is a proxy for, not a direct measurement of, the ionic egress the clinical concern actually turns on.

The decisive study therefore does not yet exist: no published work quantifies nickel or chromium release reduction (ICP-OES/ICP-MS) from a chitosan-coated orthodontic wire in oral-simulating conditions. Stating this plainly is more useful than obscuring it, because it identifies both the review’s honest limit and a concrete, tractable experiment—precisely the ICP-OES ion-release methodology already established for uncoated appliances [3,4] applied to coated ones. Until that measurement is made, ion-release mitigation for orthodontic appliances should be presented as a mechanistically plausible extrapolation, not a demonstrated property.

On biocompatibility and its assessment, chitosan coatings are consistently reported as non-cytotoxic across the cited osteoblast and fibroblast models [18,19,20,21,33,34], which is unsurprising given chitosan’s degradation to non-toxic oligosaccharides (Section 2). Two normative points are usually omitted. First, standardized evaluation is uneven: the corrosion behavior of dental metallic materials should be assessed under the methods of ISO 10271 [35], and the identification and quantification of metallic degradation products falls under ISO 10993-15 [36], yet few appliance-coating studies apply either, which is a principal source of the cross-study incomparability noted here and in the antimicrobial pillar. Second, a biodegradable barrier is a self-consuming one: as the coating hydrolyzes, its barrier function necessarily declines, and its own degradation products become part of the ISO 10993-15 biological assessment [36]. This is the point at which the ion-release argument collides with the durability problem developed in Section 5.

A biocompatibility corollary specific to composite coatings deserves explicit statement, because it changes the risk calculus rather than merely adding to it. Where the polymer alone hydrolyzes to non-toxic oligosaccharides [8,10,22], a composite matrix co-releases whatever functional agent it was loaded with as it degrades: the silver [9,26], copper [33], zinc oxide [17], or gold [18,20] species incorporated to compensate for chitosan’s modest standalone potency do not share the polymer’s benign degradation fate and several are nanoparticulate. The comparison the field has not yet made is therefore not coating versus no coating in the abstract, but the dose and toxicological profile of these released additives set against the nickel and chromium egress from an uncoated appliance that the coating is meant to suppress [3,4,31]. No orthodontic study quantifies both arms of that comparison on the same appliance; the available payload-release data are partial and context-limited—linear copper release over seven days from a NiTi implant coating [33], for instance—and none are placed beside a measured metal-ion baseline. This matters for the risk-to-benefit judgment because the two arms are not toxicologically equivalent: the uncoated baseline is dissolved metallic ions whose principal endpoint, sensitization, is already known not to track ionic dose in a simple way [6], whereas a composite coating substitutes a self-limiting polymer plus an engineered, often nanoscale, second agent whose own degradation products fall under separate biological evaluation (the ISO 10993 series, with metallic degradation products under ISO 10993-15 [36]). A composite coating can thus be claimed to improve net biological safety only once its additive-release dose has been measured against the ion release it displaces—an experiment as absent from the literature as the appliance-level ICP ion-release measurement noted above.

The defensible synthesis is narrow. Chitosan-composite coatings improve the corrosion resistance of NiTi and related alloys in vitro, chiefly on implant substrates, and one appliance-specific study shows the same electrochemical shift on molar bands (Table 2). This makes reduced ion release a reasonable expectation but not an established fact for orthodontic appliances, and any downstream reduction in allergic or biological risk is a further inference layered on top. The pillar is thus real in mechanism and thin in appliance-specific proof—an honest position that also defines the field’s most answerable open question.

## 5. Durability, Biodegradability, and Mechanical Interactions

Two problems are systematically underweighted in the optimistic coating literature, and both are decisive for clinical viability: whether the coating survives the service environment and how it alters the tribology of the appliance. Neither can be inferred from antibacterial or corrosion data, and treating them as central—rather than as caveats—is what separates a realistic appraisal from a promotional one.

The durability problem is a direct consequence of the property that makes chitosan attractive. Chitosan is enzymatically hydrolyzed in vivo, principally by lysozyme, at a rate that increases as the degree of deacetylation falls, yielding non-toxic oligosaccharides [8,10,22]. For a resorbable scaffold, this controlled degradation is the design goal; for a barrier coating expected to remain intact across an 18–24-month treatment, it is a structural liability. The concern is compounded by adhesion: chitosan bonds poorly to passivated metallic substrates, so mechanical integrity depends on substrate activation or crosslinking, and crosslinking is a genuine trade-off rather than a free improvement—it raises hydrolytic and mechanical stability at the cost of consuming the amino groups responsible for antibacterial activity, and, with some agents, of introducing cytotoxicity [20]. Critically, no appliance study characterizes coating integrity after realistic mechanical cycling or prolonged salivary exposure. The two in vivo trials run far shorter than a treatment course (six and two weeks) [23,24]; the only in vivo durability datum is Alhuwaizi’s report of unchanged device weight after aging [24], a weak proxy that does not establish retained coating coverage or function. The empirical status is therefore stark: coating longevity under combined enzymatic and mechanical load is essentially uncharacterized, and this—not antibacterial potency—is the field’s rate-limiting unknown.

A specific and answerable sub-question within this durability gap is the strength of the coating–substrate bond, since adhesion determines whether a film survives the abrasive and shear loads of daily function. Here the evidence is not merely thin but absent: no study of a chitosan coating on an orthodontic appliance reports a quantitative adhesion or bond-strength measurement—scratch/critical-load testing, pull-off strength, or nanoindentation-derived interfacial toughness—on stainless steel or NiTi substrates. The adhesion-relevant information that does exist is procedural, drawn from the electrodeposition and electrophoretic-deposition studies on implant substrates [18,19,20], which optimize deposition conditions but do not report standardized adhesion under mechanical challenge, together with the general observation that chitosan bonds poorly to passivated metal—without substrate activation or crosslinking [20]. Because tooth brushing and mastication impose exactly the repeated abrasive and shear loading that a weakly adherent, enzymatically thinning film is least equipped to withstand, coating durability under these loads cannot at present be estimated from the literature; the one in vivo durability proxy, unchanged aligner weight after aging [24], involves no abrasive challenge and cannot be extrapolated to a brushed bracket–wire surface. Closing this gap requires the pairing the field has not reported: standardized bond-strength or scratch testing and toothbrush-abrasion simulation performed before and after enzymatic aging, so that retained adhesion and coverage—not merely retained mass—are quantified over treatment-relevant loading.

The tribological problem is equally consequential and better studied, though the evidence is directionally inconsistent. Friction at the archwire–bracket interface governs sliding mechanics, anchorage control, and treatment duration; any coating necessarily alters the interfacial surface roughness and real contact area that determine it. The reported effects diverge by chemistry, substrate, and coating configuration. Chitosan and zinc-oxide nanoparticle coatings on stainless steel brackets and wires reduced frictional resistance substantially (approximately 53% for chitosan and 64% for ZnO) [37], and a ZnO nanocoating similarly lowered friction relative to conventional components [38]; silver coatings produced configuration-dependent effects, with friction lowest when only the wire was coated and surface roughness of coated components lower than uncoated before testing [39]. Against this, a silver–chitosan nanoparticle coating increased static friction and roughness on metallic brackets [26]. The correct inference is not that coatings reduce friction, but that a coating can either reduce or increase it depending on composition, filler, resulting roughness, and which component is coated—so tribological neutrality cannot be assumed and must be validated per bracket–wire system. Because friction is governed by roughness and contact mechanics rather than by antibacterial chemistry, tribological performance is not predictable from a coating’s biological performance.

These two problems are coupled, and the coupling defines the core design tension of the field. A coating optimized for antibacterial durability—thicker, crosslinked, and filler-loaded—tends toward higher roughness and worse friction; one optimized for low friction—thin and smooth—tends to degrade sooner and lose barrier function earlier. Moreover, as any coating abrades or hydrolyzes, its antibacterial and putative ion-barrier functions decline together, which means Section 3 and Section 4’s benefits are intrinsically time-limited in a manner that none of those studies quantified. No published work demonstrates a chitosan coating on an orthodontic appliance that simultaneously satisfies retained antibacterial function, corrosion barrier function, acceptable friction, and mechanical persistence over a clinically relevant interval.

The synthesis is direct: the durability–biodegradability tension, together with the unresolved friction interaction, is the principal reason clinical translation remains distant. A credible development program cannot report only zone-of-inhibition and polarization data; it must characterize coating integrity, frictional behavior, and retained antibacterial and barrier function under oral-simulating mechanical and enzymatic challenge across treatment-relevant timescales.

## 6. Clinical-Translation Perspectives

**Figure 1:**
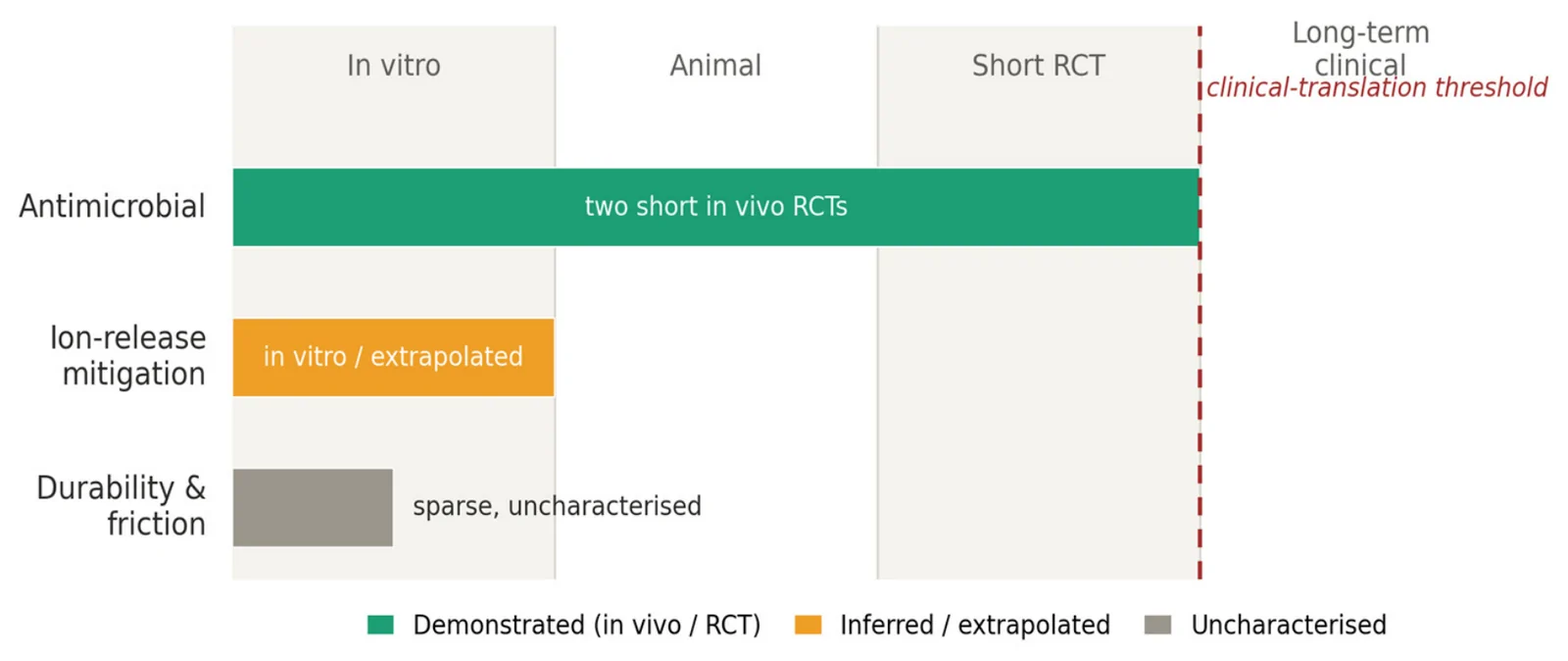
Translation-readiness map for chitosan coatings on orthodontic appliances. Each pillar is plotted by the furthest evidence stage it has reached; color encodes evidentiary strength. Antimicrobial evidence reaches short in vivo RCTs; ion-release mitigation reaches in vitro/extrapolated status; durability and friction remain sparse. No pillar has crossed the clinical-translation threshold (dashed line).

Technology-readiness for a coated appliance is set by the least-mature requirement that gates clinical use, not by the most impressive laboratory result. On that logic the concept sits at low-to-intermediate readiness: the antimicrobial pillar has reached small, short in vivo trials with surrogate endpoints [23,24]; the ion-release-mitigation pillar remains at in vitro and implant-extrapolated evidence with no appliance-level ICP measurement (Section 4); and coating durability and tribology—prerequisites for any clinical claim—are effectively uncharacterized (Section 5). A distinctive translational asset, by contrast, is chitosan’s pH-responsive antibacterial action (Section 3), which—unlike pH-independent agents such as silver—intensifies during the acidogenic episodes that drive caries; realizing this advantage clinically nonetheless remains contingent on the coating persisting long enough to deliver it. None has crossed the threshold that matters clinically, as summarized in Figure 1.

These readiness differences map directly onto distinct evidence tiers, which Table 3 sets out so that appliance-level conclusions can be separated from those extrapolated from related biomaterials.

Crossing that threshold requires studies the field has not yet run. In priority order: (i) adequately powered RCTs of treatment-length duration using WSL or caries incidence as the primary endpoint rather than microbiological surrogates [1]; (ii) coating-integrity and retained-function data under combined mechanical and enzymatic load (Section 5); (iii) per-system tribological validation, since friction effects are bracket–wire-specific and directionally inconsistent; (iv) appliance-level ICP quantification of ion-release reduction (Section 4); and (v) standardized reporting of degree of deacetylation, molecular weight, coating thickness, and crosslinking, without which cross-study synthesis remains impossible—the explicit reason the one available meta-analysis declined a pooled chitosan estimate [12]. Notably, comparator surface-prevention approaches such as peptide-based enamel coatings sit at a similar preclinical stage [40], indicating that the translational bottleneck is generic to the coating-prevention paradigm rather than specific to chitosan.

The remaining constraints are regulatory and formulational, not economic. Chitosan is inexpensive, marine-waste-derived, and depositable by scalable methods such as electrophoretic deposition [22], so cost is not the barrier. Regulation is more demanding: a coated appliance is a medical device requiring biological evaluation under the ISO 10993 series, a biodegradable coating’s own degradation products fall under ISO 10993-15 [36], corrosion behavior must be assessed per ISO 10271 [35], and EU market access additionally requires Medical Device Regulation conformity. Concretely, the development path for such a device would proceed through a biological-evaluation plan under ISO 10993-1 [41] (risk-based selection of endpoints); in vitro cytotoxicity, sensitization, and irritation testing ISO 10993-5 and -10 [42,43]; characterization and toxicological assessment of degradation products from both the alloy - ISO 10993-15 [36] and the biodegradable polymer ISO 10993-13 and -9 [44,45]; and corrosion testing per ISO 10271 [35]. In the European Union these data would feed a conformity-assessment route under the Medical Device Regulation (2017/745) [46], most plausibly for a Class IIa device, entailing Notified-Body review, a clinical-evaluation report, and a post-market clinical-follow-up plan. The practical implication is that appliance-coating studies should be designed from the outset to generate these regulatory-relevant endpoints—standardized degradation, corrosion, and biocompatibility data—rather than efficacy signals alone. The most realistic near-term configuration is therefore chitosan as a composite matrix carrying a second agent (zinc, silver, copper, or bioactive glass) rather than a standalone coating—compensating for chitosan’s modest standalone potency and finite service life, at the cost of the attribution and friction complications identified in Section 3 and Section 5.

This composite-first framing should not obscure the alternative the evidence also permits: a filler-free chitosan coating that sidesteps the nanomaterial burden entirely. A standalone configuration is not hypothetical—non-crosslinked chitosan applied directly to brackets has prevented biofilm formation without any second agent [16], confirming that the polymer carries clinically relevant intrinsic activity. Its realism for clinical use turns on a parameter window the appliance literature has not systematically explored: a degree of deacetylation high enough to maximize the protonatable glucosamine groups responsible for activity yet low enough to slow lysozyme-mediated hydrolysis to a treatment-compatible rate (the DD–durability trade-off of Section 2 and Section 5); a molecular weight high enough for antibacterial performance [7] but compatible with uniform deposition and adhesion; and a thickness thin enough for tribological neutrality yet sufficient for service life. Because studies almost never report this degree-of-deacetylation/molecular-weight/thickness triplet [12], the optimal filler-free formulation cannot yet be specified from existing data—but the regulatory argument favors resolving it. A standalone chitosan coating avoids the nanoparticle-specific scrutiny and the additional degradation-product evaluation [36] that incorporated silver, copper, zinc, or gold nanophases would trigger, trading a lower efficacy ceiling and shorter service life for a markedly simpler path to conformity. The defensible position is therefore that a filler-free chitosan coating is the most regulatorily tractable near-term option and is clinically plausible on current mechanistic evidence, but that establishing the degree of deacetylation, molecular weight, and thickness that would make it durable and effective is itself one of the field’s unmet experimental needs,which Table 4 sets out together with the studies required to close them.

## 7. Conclusions

Chitosan-based coatings for orthodontic appliances are a mechanistically coherent but evidentially uneven proposition, and the three pillars named in this review’s title are not equally supported. The antimicrobial pillar is the empirical core: chitosan and chitosan-composite coatings consistently reduce bacterial colonization of brackets, bands, archwires, and aligners in vitro (though direct evidence for chitosan alone on an orthodontic appliance remains limited to a single bracket study [16], with most reports evaluating chitosan–nanoparticle composites), and two short randomized trials now extend this to the mouth—though all evidence rests on microbiological surrogates rather than the white spot lesion outcome that motivates the field, and the polymer’s independent contribution is rarely isolated from co-deposited fillers. The ion-release-mitigation pillar is mechanistically plausible but not demonstrated for appliances: chitosan-composite coatings improve the corrosion resistance of NiTi and related alloys, yet this evidence is drawn chiefly from implant substrates and rests on electrochemical proxies, and no study has quantified reduced metal-ion release from a chitosan-coated orthodontic wire under oral-simulating conditions. The clinical-translation pillar is, candidly, a roadmap rather than a result.

The rate-limiting problem is neither antimicrobial potency nor corrosion theory but durability: chitosan’s biodegradability, the property underwriting its biocompatibility, undermines the persistence a barrier coating requires across an 18–24-month treatment, and its interaction with sliding mechanics remains under-characterized. Therefore, progress hinges not so much on further proving in vitro efficacy but on filling the gaps—treatment-length clinical endpoints, coating integrity and retained-function data, appliance-level ion-release measurement, and standardized reporting. The economic factor coupled with the non-toxic source of chitosan makes it an attractive solution, most likely as a composite material, but its use as a clinical coating is dependent on evidence that is readily achievable but currently lacking.

## Figures and Tables

**Table 1:** Antimicrobial evidence for chitosan-based coatings on orthodontic appliances.

Study	Appliance	Coating (Method)	Model	Organism(s)	Key Outcome	Tier
Patel 2026 [23]	NiTi archwire	Chitosan NPs (ionic gelation)	In vivo split-mouth RCT (n = 30; 6 wk)	*S. mutans*; *L. acidophilus*	CFU ↓ ≈93% and ≈98% vs. uncoated (*p* < 0.001)	RCT (surrogate)
Alhuwaizi 2025 [24]	3D-printed aligner	3% chitosan NPs (matrix)	In vivo RCT (N = 32; 1–2 wk)	*S. mutans* (ATCC + clinical)	CFU ↓ (effect size, ES > 0.9); PI and BOP ↓; weaker vs. clinical isolate	RCT (surrogate)
Wang Y 2023 [16]	Bracket	Non-crosslinked chitosan (dip/freeze-dry)	In vitro	*S. mutans* biofilm	Dose-dependent biofilm inhibition 48.5 → 99.6%	In vitro (isolates chitosan)
Fatima 2025 [17]	Molar band	Chitosan–ZnO (EPD)	In vitro	*S. mutans*	Significant inhibition; +corrosion resistance (see Section 4)	In vitro
Liu 2024 [25]	Bracket	CMC–xylitol carbon dots (PDA-adhered)	In vitro + murine	*S. mutans*	≈80% antibacterial; +remineralization; cytocompatible	In vitro + animal
Tawakal 2023 [26]	Bracket (ceramic/metallic)	Ag/Ag–chitosan NPs	In vitro	*S. mutans*	Significant inhibition; ↑ friction/roughness on metal	In vitro

Non-chitosan comparator coatings (Ag, ZnO, ZrO_2_, TiO_2_) are discussed in the text [12,27,28,29] and excluded from this table to preserve chitosan-specific scope. NPs, nanoparticles; EPD, electrophoretic deposition; PI, plaque index; BOP, bleeding on probing; ES, effect size; CMC, carboxymethyl chitosan; PDA, polydopamine.

**Table 2:** Corrosion, ion-release, and biocompatibility evidence for chitosan-based (and comparator) coatings.

Study	Substrate	Coating (Method)	Endpoint	Result	Context/Flag
Mikulewicz 2024 [3]	SS, NiTi, CrCo, TMA wire (uncoated)	-	Ni/Cr release, ICP-OES, artificial saliva, 4 wk	NiTi and CrCo > SS for Ni/Cr	Orthodontic—baseline problem
Fatima 2025 [17]	Orthodontic band	Chitosan–ZnO (EPD)	Potentiodynamic polarization	Ecorr −0.6966 → −0.5949 V; icorr 7.81 → 4.04 µA/cm^2^	Orthodontic appliance—direct
Chen 2023 [32]	NiTi archwire	PDA–GO (self-assembly; non-chitosan)	Ni dissolution; cytocompatibility	Reduced Ni dissolution; cytocompatible	Orthodontic—non-chitosan comparator
Jabłoński 2026 [33]	NiTi	Chitosan/Cu-NP multilayer (immersion + IGC)	Corrosion current density; Cu release; cytotox; antibacterial	↓ corrosion current density (value pending full text); non-cytotoxic MG-63; linear Cu release; kills *E. coli*	Implant—extrapolated
Yang 2025 [34]	LPBF NiTi	DCPD–PMTMS–chitosan (electrodeposition + dip)	Corrosion current density; viability; antibacterial	↓ corrosion current density (value pending full text); viability 108%; >70% antibacterial	Implant—extrapolated
Farghali 2015 [18]	Titanium	Chitosan–Au-NP (electrodeposition)	Corrosion (EIS/polarization), Hanks’	Inhibition efficiency ≈98%; antibacterial	Implant—extrapolated
Rajabinezhad 2025 [19]	316L SS	Cu–Fe3O4/bioglass/chitosan (EPD)	Corrosion; antibacterial; ISO 10993-5	Improved corrosion resistance; cytocompatible	Implant—extrapolated
Bartmański 2025 [20]	Ti alloy	Chitosan–nanogold/nanozinc (EPD)	Adhesion/mechanical; biological	Coating integrity and biological response characterized	Implant—extrapolated

No row reports ICP-quantified ion-release reduction from a chitosan-coated orthodontic appliance—the field’s principal evidence gap. SS, stainless steel; TMA, titanium–molybdenum alloy; PDA–GO, polydopamine–graphene oxide; IGC, inert gas condensation; LPBF, laser powder-bed fusion; Ecorr, corrosion potential; icorr, corrosion current density.

**Table 3:** Hierarchy of evidence underlying the three pillars, from appliance-level in vitro data to true orthodontic clinical endpoints. The table distinguishes conclusions directly supported by appliance-level evidence from those extrapolated from related biomaterial studies.

Evidence Tier	Representative Evidence in This Review	What It Can and Cannot Support
In vitro appliance studies (brackets, bands, archwires, aligners)	CFU/biofilm assays and electrochemical corrosion tests on the actual device [16,17,25,26,32]	Establish surface antibacterial activity and, in one case, improved corrosion behavior on an appliance; cannot demonstrate caries or sensitisation outcomes, nor in-service durability.
Short-term in vivo surrogate outcomes	Two RCTs measuring adherent CFU, plaque and bleeding indices over 2–6 weeks [23,24]	Confirm an intraoral antibacterial effect at the appliance surface; endpoints are microbiological surrogates over a small fraction of treatment length, not white-spot-lesion or caries incidence.
Implant-derived/tissue-engineering corrosion studies	Chitosan-composite coatings on NiTi and related alloys, chiefly non-orthodontic substrates [18,19,20,21,33,34]	Support the corrosion-barrier mechanism by extrapolation; substrate, geometry and loading differ from orthodontic wires, so appliance-level ion-release reduction remains inferred rather than measured.
True orthodontic clinical endpoints	WSL/caries incidence, nickel sensitisation, ICP-quantified ion release from a coated appliance—none identified	The outcomes that would justify clinical claims; not yet studied for chitosan-coated orthodontic appliances.

**Table 4:** Translational evidence gaps and the studies required to close them.

Domain	Current Status	Required Study
Clinical efficacy	Surrogate CFU/biofilm endpoints; ≤6-wk exposure [23,24]	Treatment-length RCT with WSL/caries as primary endpoint
Coating durability	Uncharacterized under oral load; weight proxy only [24]	Integrity + retained-function testing under mechanical/enzymatic cycling
Tribology	Direction-dependent; chitosan data ≈ 2 points [26,37]	Per-bracket–wire-system friction validation
Ion-release mitigation	Corrosion proxies; implant-extrapolated [17,33,34]	Appliance-level ICP-OES/ICP-MS release quantification [3,4]
Standardization	DD/MW/thickness under-reported [12]	Consensus reporting minimum for coating studies
Regulatory	No device pathway addressed	ISO 10993/10271 evaluation; MDR conformity route [35,36]

## Data Availability

The original contributions presented in the study are included in the article, further inquiries can be directed to the corresponding author.

## Abbreviations

The following abbreviations are used in this manuscript:

BOP	Bleeding on probing
CFU(s)	Colony-forming unit(s)
CMC	Carboxymethyl chitosan
Cr–Co	Chromium–cobalt (alloy)
DD	Degree of deacetylation
Ecorr	Corrosion potential
EPD	Electrophoretic deposition
ES	Effect size
EU	European Union
icorr	Corrosion current density
ICP-MS	Inductively coupled plasma–mass spectrometry
ICP-OES	Inductively coupled plasma–optical emission spectroscopy
IGC	Inert gas condensation
ISO	International Organization for Standardization
LPBF	Laser powder-bed fusion
MW	Molecular weight
NiTi	Nickel–titanium (alloy)
NPs	Nanoparticles
PDA	Polydopamine
PDA–GO	Polydopamine–graphene oxide
PI	Plaque index
RCT(s)	Randomized controlled trial(s)
SANRA	Scale for the Assessment of Narrative Review Articles
SS	Stainless steel
TMA	Titanium–molybdenum alloy
WSL(s)	White spot lesion(s)
